# Prevalence of Transthyretin Amyloid Cardiomyopathy Among Acute Heart Failure Patients with Hypertrophy Across the Left Ventricular Ejection Fraction Spectrum

**DOI:** 10.3390/jcm13237103

**Published:** 2024-11-24

**Authors:** Maria Velliou, Lampros Markos, Stella Qiuris, Sofia Bezati, Ioannis Ventoulis, Dionysis Matsiras, Vasiliki Bistola, Ignatios Ikonomidis, Effie Polyzogopoulou, John T. Parissis

**Affiliations:** 1 University Department of Emergency Medicine, Attikon University Hospital, National and Kapodistrian University of Athens, 12462 Athens, Greece; 2 Department of Occupational Therapy, University of Western Macedonia, 50200 Ptolemaida, Greece; 3 Second Department of Cardiology, Attikon University Hospital, National and Kapodistrian University of Athens, 12462 Athens, Greece

**Keywords:** acute heart failure, left ventricular hypertrophy, transthyretin amyloid cardiomyopathy, ATTR, emergency department, cardiac biomarkers

## Abstract

**Background/Objectives:** Transthyretin amyloid (ATTR) cardiomyopathy mimics left ventricular hypertrophy (LVH) and has been identified as a specific cause of heart failure (HF). The aim of this study was to assess the prevalence of ATTR among patients presenting to the Emergency Department (ED) with acute HF (AHF) and LVH and explore their clinical characteristics and outcomes. **Methods:** Of 127 AHF patients with LVH, 95 completed the diagnostic protocol, which included monoclonal paraprotein testing and technetium-99 m pyrophosphate scintigraphy. Patients were followed for 6 months, and adverse events, including mortality and HF-related hospitalizations, were recorded. **Results:** ATTR was diagnosed in 8.4% of patients. The mean left ventricular ejection fraction (EF) was 46 ± 7% in ATTR subjects, with 25% classified as HF with reduced EF, 37.5% HF with mildly reduced EF, and 37.5% HF with preserved EF. N-terminal pro b-type natriuretic peptide (NT-proBNP) and high sensitivity troponin T (hs-TnT) were higher in ATTR compared to the non-ATTR group [NT-proBNP: 5863 (6519–12382) pg/mL versus 3586 (1393.5–6322) pg/mL, *p* = 0.007; hs-TnT: 35.9 (47.9–83.8) pg/mL versus 30.0 (19.4–49.5) pg/mL, *p* = 0.0006]. During follow-up, twenty-three patients from the cohort died: six in the ATTR and seventeen in the non-ATTR group. The estimated survival rate was significantly lower in ATTR versus non-ATTR patients (log-rank *p* < 0.0001). **Conclusions:** In this cohort of AHF patients with LVH presenting to the ED, ATTR cardiomyopathy was detected in 8.4%. Using routinely used cardiac biomarkers and basic echocardiography allows for the raising of suspicion of the disease from the ED setting, potentially facilitating earlier diagnosis in this population.

## 1. Introduction

Left ventricular (LV) hypertrophy (LVH) is defined as increased LV wall thickness and is classified either as primary due to mutations in sarcomere protein genes or accumulation of abnormal substances in the myocardium (pseudohypertrophy) or as secondary in response to long-term volume or pressure overload [1]. LVH is present in approximately 15% of males and 9% of females in the general population and is an independent risk factor for heart failure (HF), owing to the fact that LVH increases LV myocardial stiffness and impairs relaxation, which, in turn, leads to increased LV end-diastolic and pulmonary venous pressures [2,3,4].

Echocardiography is the first-line imaging modality for the detection of LVH because of its wide availability in different settings [5]. Detection of LVH in echocardiography should raise the suspicion of infiltrative cardiomyopathies, of which transthyretin amyloid (ATTR) cardiomyopathy is an increasingly recognized cause of HF among elderly patients with LVH. In patients aged ≥60 years who are admitted to the hospital for HF with preserved ejection fraction (HFpEF), its prevalence has been reported to exceed 13% [6]. In ATTR, cardiac structures are infiltrated by insoluble amyloid fibrils consisting of destabilized transthyretin monomers. Because ATTR resembles other cardiac conditions, predominantly hypertrophic cardiomyopathy and HF, its diagnosis is frequently delayed, and so is the initiation of appropriate treatment [7]. Given the availability of specific therapies that slow disease progression, prompt diagnosis of ATTR is of paramount importance [8].

Advanced echocardiography, particularly myocardial deformation imaging and myocardial work assessment, represent novel methods that may help to discriminate between different hypertrophic phenocopies. Myocardial work indexes, such as the global work index (GWI) and global constructive work (GCW), are more impaired in ATTR subjects compared to those with hypertrophic cardiomyopathy and hypertension [9,10]. A recently published study of 60 HFpEF patients (30 with nonobstructive hypertrophic cardiomyopathy and 30 with ATTR) showed that both systemic vascular resistance and GWI were impaired in 43% of patients with hypertrophic cardiomyopathy and 93% of ATTR counterparts [11]. Even though transthoracic echocardiography provides useful insights into the evaluation of cardiac anatomy and function, it lacks specificity and the ability to characterize myocardial tissue. Poor imaging quality in several cases, such as in obese or aged patients, imposes the need to incorporate additional imaging modalities for the diagnosis of ATTR [12].

The aim of the present study was to explore the prevalence of ATTR among patients who present to the Emergency Department (ED) with acute HF (AHF) and LVH on echocardiography, as well as to assess their clinical, echocardiographic, and laboratory characteristics and short-term outcomes when compared to patients with AHF and LVH due to other etiologies (non-ATTR patients).

## 2. Materials and Methods

Study population. This is a single-center prospective observational study that included patients presenting to the ED of Attikon University Hospital in Athens, Greece, between February 2022 and December 2023, with signs and/or symptoms of AHF, who were classified as New York Heart Association (NYHA) class II-IV and had LVH on 2-dimensional (2D) echocardiography, irrespective of LV ejection fraction (LVEF). A written informed consent was obtained from each participant. Patients with acute coronary syndrome or refusal to provide informed consent due to unwillingness to participate in the research were excluded from the study. The current study was conducted in accordance with the Declaration of Helsinki and was approved by the Hospital Ethics Committee.

Upon enrolment, a detailed medical history, including demographics (age, gender) and comorbidities (arterial hypertension, dyslipidemia, diabetes mellitus, coronary artery disease, atrial fibrillation, valvulopathies), was obtained from each patient. Initial assessment included physical examination, electrocardiogram (ECG), chest X-ray, and transthoracic 2D echocardiography. Venous blood samples were collected for standard biochemistry and cardiac biomarkers, including N-terminal pro b-type natriuretic peptide (NT-proBNP) and high-sensitivity cardiac troponin T (hs-TnT). All patients underwent diagnostic evaluation for ATTR with monoclonal paraprotein testing, which included serum free light chain assay for kappa and lambda chains, as well as serum and urine immunofixation. Moreover, the patients underwent technetium-99 m pyrophosphate (99mTc-PYP) scintigraphy, which was performed after their clinical stabilization. The PYP scan was evaluated using a previously reported visual grading scale of myocardial radiotracer uptake that ranged from 0 to 3 (grade 0: no cardiac uptake, grade 1: cardiac uptake less than bones, grade 2: cardiac uptake equal to bones, grade 3: cardiac uptake greater than bones) [13,14]. The scan was considered positive when it revealed a moderate or severe uptake (score 2 or 3) in both ventricles. Patients with excessive blood pool activity, a common cause of a false positive PYP scan, were characterized as non-ATTR. ATTR diagnosis was confirmed if the PYP scan was positive and monoclonal paraprotein testing was normal.

The patients were followed for 6 months after ED presentation and clinical events, including death and HF-related rehospitalizations, which were recorded.

Echocardiography. All patients underwent transthoracic 2D echocardiography. LVH was defined as interventricular septum (IVS) thickness ≥12 mm and/or LV posterior wall (LVPW) thickness ≥12 mm. LV end-diastolic and end-systolic diameters, LVEF, right ventricular (RV) basal diameter at end-diastole, left atrial (LA) size, and LV diastolic dysfunction were also measured. LV diastolic dysfunction was evaluated by means of transmitral flow velocity patterns on pulsed wave Doppler, mitral annular velocities on tissue Doppler imaging, tricuspid regurgitant (TR) systolic jet velocity on continuous wave Doppler, and LA maximum volume index.

Statistical analysis. Continuous variables were expressed as mean values ± standard deviation (SD) and categorical variables as absolute numbers and percentages. Laboratory results were expressed as median with interquartile range (IQR, 25th–75th). Comparisons of differences between ATTR and non-ATTR groups were performed using a t-test for quantitative variables and a chi-square test for qualitative variables. Overall survival was described using Kaplan–Meier curves, and a log-rank test was used to determine if there were differences in the survival distribution between ATTR and non-ATTR. All tests were two-tailed, and *p*-values < 0.05 were considered statistically significant.

## 3. Results

**Clinical and echocardiographic characteristics.** Between February 2022 and December 2023, 127 patients presented to the ED with signs and/or symptoms of AHF and LVH. Of the 127 patients, 35 did not complete the study protocol, resulting in a total number of 95 patients undergoing final analysis.

The mean age of the final study population was 72 ± 14 years, while 66% of the patients were males. Regarding comorbidities, 72% had hypertension, 60% had dyslipidemia, 38% had valvulopathies, 30% had diabetes mellitus, 26% had atrial fibrillation, and 6% had coronary artery disease. Moreover, 58% reported a past history of HF hospitalization (Table 1).

ECG analysis revealed sinus rhythm without ischemic changes in the vast majority of patients. AF was detected in twenty-five patients, low QRS voltage in one patient, and pseudoinfarction pattern in one patient. Chest X-ray revealed pleural effusion in 35 patients.

Echocardiographic analysis showed that LVEF in the total patient cohort was 45 ± 11%, with 48% of patients being classified as HF with reduced EF (HFrEF), 16% as HF with mildly reduced EF (HFmrEF), and 36% as HFpEF. Diastolic dysfunction was evident in 87% of the patients (Table 2).

Management and clinical outcomes. Forty-seven (49.5%) patients received loop diuretics, while fifteen (16%) received vasodilators (nitrates). Noninvasive mechanical ventilation was used in five (5.3%) patients in the ED. Fifty-nine (62%) patients were admitted to the hospital, whereas thirty-six (38%) patients were discharged from the ED. The majority of admitted patients were hospitalized in the cardiology ward. Only three patients were admitted to the coronary care unit (CCU) and thirteen patients to the internal medicine ward due to coexisting lower respiratory tract or urinary tract infections. Admissions to the CCU were due to acute pulmonary edema and/or the need for mechanical ventilatory support. The mean hospital length of stay was 6.0 ± 3.3 days. During the 6-month follow-up period, 28 patients (24.2%) were re-admitted due to decompensated HF or anemia, while 23 (29.5%) patients died (Table 3).

ATTR cardiac amyloidosis. ATTR was diagnosed in eight patients (8.4%) and all of them had a PYP grade 3. Their mean age was 86 ± 5 years, with the majority (87.5%) being males. With regard to comorbidities, four patients had arterial hypertension, three had dyslipidemia, four had atrial fibrillation, and one was diabetic.

The echocardiographic assessment showed that LVEF was 46 ± 7% in ATTR patients, and there was no significant difference compared to non-ATTR patients (Table 2). Two (25%) patients with ATTR were classified as HFrEF, three (37.5%) as HFmrEF, and three (37.5%) as HFpEF. Compared to their non-ATTR counterparts, ATTR patients had smaller LV end-diastolic diameter (*p* = 0.03), greater LVPW thickness (*p* = 0.01), and larger RV basal diameter at end-diastole (*p* = 0.01), whereas no significant differences were observed in LV end-systolic and LA diameters (Table 2).

Admission levels of NT-proBNP were higher in ATTR compared to non-ATTR patients [5863 (6519–12382) pg/mL vs. 3586 (1393.5–6322) pg/mL respectively, *p* = 0.007]. Likewise, ATTR patients had higher levels of hs-TnT than non-ATTR patients [35.9 (47.9–83.8) pg/mL versus 30.0 (19.4–49.5) pg/mL respectively, *p* = 0.0006].

**Figure 1:**
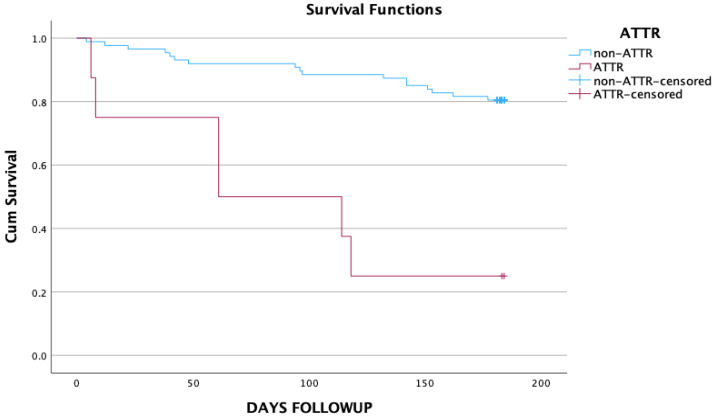
Kaplan–Meier curves depicting overall survival in ATTR and non-ATTR groups. ATTR: transthyretin amyloid cardiomyopathy.

During follow-up, six out of eight ATTR patients died (Table 3). Kaplan–Meier analysis revealed that the overall survival rate of patients with ATTR cardiomyopathy was significantly lower than their non-ATTR counterparts (log-rank chi-square 20.419, *p* < 0.0001) (Figure 1).

## 4. Discussion

To our knowledge, this is the first study to provide data about the prevalence of ATTR cardiomyopathy among patients presenting to the ED with AHF and morphological features of LVH, irrespective of the LVEF phenotype. The prevalence of ATTR cardiomyopathy was found to be approximately 9%. Compared to their non-ATTR counterparts, ATTR patients were older, more frequently males, and with higher natriuretic peptide and troponin levels. Moreover, they showed greater LVPW thickness with smaller LV cavity size and larger RV dimensions, albeit with similar LVEF.

Cardiac amyloidosis has been identified as a frequently underdiagnosed cause of HF. The two most common types are light chain immunoglobulin (AL) and ATTR cardiac amyloidosis. Regarding ATTR, its true prevalence is currently unknown. It is estimated that ATTR cardiomyopathy may be present in approximately 6–16% of patients aged above 65 years with unexplained LVH, HFpEF, or severe aortic stenosis [15,16]. The AC-TIVE study is an Italian nationwide survey, which was conducted in two phases and investigated the prevalence and diagnostic accuracy of echocardiographic red flags of cardiac amyloidosis in a cohort of patients undergoing routine echocardiography without any prior clinical suspicion. Phase I revealed that at least one echocardiographic red flag was present in 7% of patients aged ≥55 years. Phase II showed that, after performing bone scintigraphy and monoclonal paraprotein testing, 29% received the final diagnosis of cardiac amyloidosis (ATTR cardiac amyloidosis: 24%, AL cardiac amyloidosis: 5%) [17,18]. Another study also found that in a population of 286 patients with HFpEF and thickened ventricular walls, ATTR was present in 6% [19]. Moreover, in a recently published study of 100 consecutive adults with unexplained increased LV wall thickness, it was reported that 12% were diagnosed with ATTR [20]. In the current cohort of AHF patients with LVH presenting to the ED, the prevalence of ATTR was 8.4%. Therefore, it is important for the ED physician to maintain increased awareness of the possibility of amyloidosis in patients presenting with unexplained increased LV wall thickness in order to reach an early diagnosis of cardiac amyloidosis and initiate prompt treatment.

Although ATTR cardiomyopathy is predominantly considered to manifest as HFpEF, one-third of patients with ATTR cardiac amyloidosis actually exhibit LVEF < 50% at the time of diagnosis due to the progressive infiltrative nature of the disease [21]. A previous study, which included ATTR patients, has reported that at the time of diagnosis, 31% of patients were classified as HFrEF (LVEF ≤ 40%) and 69% as HFmrEF/HFpEF (LVEF ≥ 40%) [22]. Likewise, a registry of 213 consecutive ATTR patients from the Cleveland Clinic Florida Amyloid Center confirmed the heterogeneity across the LVEF spectrum since 21.6% presented with HFrEF, 17.8% with HFmrEF, and 60.6% with HFpEF upon diagnosis [23]. Similarly, in the current study, ATTR patients had LVEF values spanning the entire spectrum of LV systolic function, with one-quarter of the patients having HFrEF, and over one-third of them each having either HFmrEF or HFpEF. These findings are in contrast to the traditional association of ATTR with the HFpEF phenotype alone. It is possible that the reduction of LVEF might be secondary to a longer duration of comorbidities, such as hypertension or ischemic heart disease, or might be merely related to more advanced stages of cardiac amyloidosis.

ATTR is characterized by a remarkable male predominance, while its incidence increases significantly with age. Available data indicate that up to 25% of patients with ATTR cardiomyopathy are over 80 years old [24]. In our study, almost 90% of the ATTR patients were men aged over 80 years, in line with the previous literature.

Cardiac biomarkers, including natriuretic peptides and troponins, are not only useful adjunctive diagnostic markers of cardiac involvement in amyloidosis but also significant predictors of its clinical course. Indeed, their levels are often increased disproportionately to the severity of cardiac systolic and/or diastolic dysfunction in ATTR patients [25]. A prospective study of 120 HFpEF patients, among whom 16 had ATTR, showed that NT-proBNP and ultrasensitive troponin I were significantly higher in ATTR compared to undifferentiated HFpEF [6]. According to our study, the same applies to ATTR patients who present to the ED with acute decompensated HF when compared to non-ATTR patients with AHF and LVH due to other etiologies. Therefore, the role of NT-proBNP and hs-TnT as supplementary diagnostic tools for cardiac amyloid involvement might also be relevant in the AHF setting.

The current registry showed that the majority of patients presenting to the ED with AHF and LVH were not related to ATTR but instead had a high burden of cardiovascular comorbidities, predominantly arterial hypertension, which is actually the most common cause of LVH. A meta-analysis of 30 studies that included 37,700 hypertensive patients showed that the prevalence of LVH ranged from 36% to 41% among hypertensive patients and further increased in those with severe hypertension, type-2 diabetes mellitus, a history of previous cardiovascular events, or refractory hypertension [26]. Also, pre-hypertension seems to be associated with structural and functional cardiac changes. A meta-analysis of almost 74,000 untreated subjects, who were normotensive, hypertensive, or pre-hypertensive, demonstrated that LV mass index and relative wall thickness were greater in individuals with pre-hypertension when compared to normotensive participants, but definitely smaller when compared to hypertensive subjects [27].

The echocardiographic assessment of LA function seems to be of paramount importance to the differential diagnosis of patients with hypertrophic phenotypes. In cardiac amyloidosis, LA dysfunction is manifested by LA enlargement, increased LA stiffness, and decreased LA contractility [28]. A retrospective, observational study of 100 patients (33 with ATTR, 34 with hypertrophic cardiomyopathy, and 33 controls) revealed the potential supportive role of speckle-tracking echocardiography in the early detection of the disease. All atrial strain parameters were significantly lower in ATTR subjects, including LA-reservoir, LA-conduit, and LA-contraction [29].

Although echocardiography remains the first-line non-invasive screening tool in raising suspicion of cardiac amyloidosis and serves as a gatekeeper for further diagnostic tests, it lacks specificity. Typical echocardiographic findings might not be present in the early stage of the disease, making discrimination within the context of hypertrophic phenotypes difficult. On the other hand, diastolic dysfunction might occasionally be evident before LV wall thickness increases [30]. A study of 544 patients with cardiac amyloidosis and 200 controls (patients with clinically suspected cardiac amyloidosis but negative diagnostic tests) showed that the apical sparing pattern in longitudinal strain, which is considered a hallmark echocardiographic feature on cardiomyopathies [12], was 72% sensitive and 66% specific, even when used the lower optimal cut-off of 1.67 [31].

Cardiac magnetic resonance (CMR) offers additional diagnostic clues for the suspicion of cardiac amyloidosis, most importantly regarding myocardial tissue characterization. Typical morphological features in CMR include concentrical pseudo-hypertrophy, thickened left atrial wall and interatrial septum, and left atrial enlargement. The Late Gadolinium Enhancement technique reveals the low difference in signal intensities between the LV cavity and blood pool. Native T1 mapping and extracellular volume are also elevated in cardiac amyloidosis, and both parameters are correlated with high diagnostic accuracy [32,33].

Recently, technetium-labeled cardiac scintigraphy using bone-specific radiotracers (PYP, DPD, HMDP) has enabled non-biopsy diagnosis of ATTR because of its high diagnostic accuracy. However, there are specific caveats that need to be avoided in order to avoid misdiagnoses [34]. In particular, false positive results might be observed in light-chain amyloidosis (AL), which necessitates concurrent monoclonal paraprotein testing to exclude AL. Another cause of false positive scans is excessive blood pool activity, which is avoided by applying single-photon emission computed tomography (SPECT) along with planal imaging, as applied in the present study.

There is evidence that the underlying comorbidities might have a prognostic impact on hypertrophic cardiomyopathy [35]. A retrospective study of 109 individuals with hypertrophic cardiomyopathy showed that the underlying comorbidities were hypertension (63.3%), obesity (78%), coronary artery disease (31.2%), diabetes mellitus (22.0%), atrial fibrillation (12.8%), and stroke (4.6%) [36]. In the present study, the comorbidities of patients with LVH were hypertension (72%), dyslipidemia (60%), valvulopathies (38%), diabetes mellitus (30%), atrial fibrillation (26%), and coronary artery disease (6%). In the ATTR population, four patients had arterial hypertension, three had dyslipidemia, four had atrial fibrillation, and one had diabetes mellitus.

Diagnosis of ATTR amyloidosis is a multistep process and delays or misdiagnoses may result in cumulative organ damage, poor prognosis, and increased mortality. Available evidence indicates that the hospital readmission rate for any cause, including AHF, is higher in HF patients with a known history of cardiac amyloidosis [37]. Therefore, the combined use of cardiac biomarkers and echocardiography by allowing early screening as soon as a patient presents to the ED may raise suspicion of the disease, even in the emergency setting, and potentially contribute to the timely initiation of ATTR-specific therapies shown to improve outcomes and quality of life.

## 5. Limitations

The present study has some limitations. First, it was a single-center study. Second, the number of recruited patients was relatively small, probably due to the frailty of the screened population. The limited number of participants reduces the power of the study and makes it difficult to extrapolate the results. Thus, findings should be replicated in larger cohorts to ensure their reliability and generalizability.

## 6. Conclusions

In the setting of AHF, across the whole spectrum of LVEF, ATTR was diagnosed as the underlying cause of cardiomyopathy in 8.4% of patients who presented to the ED with morphological features of LVH on 2D echocardiography. Given that ATTR cardiomyopathy is a rare and often under-recognized disease, the diagnosis is challenging and requires a high index of clinical suspicion. Initiation of screening for ATTR appears to be feasible as soon as the patient presents to the ED. This early strategy might expedite the process of disease diagnosis, which will, in turn, facilitate prompt treatment initiation. In this context, both echocardiography and cardiac biomarkers, including natriuretic peptides and troponin, measured on admission may retain their potential as important screening tools for the early suspicion of ATTR in the AHF setting. ATTR patients with AHF have worse short-term survival rates than their non-ATTR counterparts, thereby emphasizing the need for prompt diagnosis and treatment.

## Figures and Tables

**Table 1:** Baseline characteristics of the study population.

Demographics	All Patients (n = 95)	Non-ATTR (n = 87)	ATTR (n = 8)	*p*-Value
Age (years)	72 ± 14	72 ± 11	86 ± 5	0.0001
Male sex, n (%)	63 (66.3%)	56 (64.4%)	7 (87.5%)	0.06
NYHA class, n (%)				
II	56 (59%)	53 (61%)	3 (37.5%)	0.13
III	24 (25%)	22 (25%)	2 (25%)	0.42
IV	15 (16%)	12 (14%)	3 (37.5%)	0.14
Comorbidities, n (%)				
Hypertension	68 (72%)	64 (74%)	4 (50%)	0.13
Diabetes mellitus	28 (30%)	27 (31%)	1 (12.5%)	0.10
Dyslipidemia	57 (60%)	54 (62%)	3 (37.5%)	0.12
Coronary artery disease	6 (6%)	6 (7%)	0	0.01
Valvulopathies	36 (38%)	30 (35%)	6 (75%)	0.02
Atrial Fibrillation	25 (26%)	21 (24%)	4 (50%)	0.11
Previous HF hospitalizations	55 (58%)	50 (58%)	5 (62.5%)	0.40
HF type, n (%)				
HFrEF	46 (48%)	44 (50%)	2 (25%)	0.09
HFmrEF	15 (16%)	12 (14%)	3 (37.5%)	0.12
HFpEF	34 (36%)	31 (36%)	3 (37.5%)	0.46

ATTR: transthyretin amyloid cardiomyopathy; HF: heart failure; HFmrEF: heart failure with mildly reduced ejection fraction; HFpEF: heart failure with preserved ejection fraction; HFrEF: heart failure with reduced ejection fraction; NYHA: New York Heart Association.

**Table 2:** Echocardiographic and laboratory data of the study population.

Parameters	Total (n= 95)	Non-ATTR (n = 87)	ATTR (n = 8)	*p*-Value
Echocardiographic data				
LVEF (%)	45.4 ± 11.1	45.3 ± 11.6	46.2 ± 7.4	0.4
LVEDD (mm)	50.3 ± 9.6	51.1 ± 9.8	45.1 ± 6.2	0.03
LVESD (mm)	36.7 ± 9.7	37.1 ± 10.1	33.6 ± 6.3	0.2
IVS (mm)	12.6 ± 1.5	12.5 ± 1.6	13.1 ± 1.6	0.3
LVPW (mm)	12.3 ± 1.0	12.1 ± 0.9	13.4 ± 1.1	0.01
LA diameter (mm)	47.0 ± 6.4	47.0 ± 6.6	47.1 ± 5.3	0.9
RV basal diameter (mm)	32.6 ± 6.4	31.8 ± 6.1	37.6 ± 5.8	0.01
Diastolic dysfunction (n, %)	83 (87.4%)	77 (88.5%)	6 (75%)	0.2
Grade I	40 (42.1%)	39 (44.8)	1 (12.5%)	
Grade II	24 (25.3%)	21 (24.1%)	3 (37.5%)	
Grade III	17 (17.9%)	15 (17.3%)	2 (25%)	
Grade IV	2 (2.1%)	2 (2.3%)	-	
*Laboratory data*				
Hematocrit (%)	31.1 (32.6–40.9)	36.1 (32.8–41.3)	35.5 (31.2–38.7)	0.1
Hemoglobin (g/dL)	11.9 (10.8–13.2)	11.9 (10.8–13.5)	11.7 (10.3–12.7)	0.3
Urea (mg/dL)	58.9 (42.8–85.3)	56.6 (40.0–80.3)	79.1 (59.4–118.9)	0.02
Creatinine (mg/dL)	1.1 (0.8–1.6)	1.1 (0.8–1.6)	1.4 (1.1–1.8)	0.3
MDRD-based eGFR (mL/min/1.73 m^2^)	59.2 (37.2–80.9)	60.6 (39.4–81.5)	47.5 (35.7–62.5)	0.03
SGOT (U/L)	21.0 (16.0–31.0)	21.0 (15.5–31.0)	26.5 (20.0–35.0)	0.1
SGPT (U/L)	18.5 (12.0–27.0)	18.5 (12.0–28.0)	18.5 (13.0–22.0)	0.06
Sodium (mmol/L)	139.0 (136.0–141.0)	139.0 (136.5–141.0)	138.0 (134.0–138.5)	0.2
Potassium (mmol/L)	4.4 (4.1–4.8)	4.3 (4.1–4.7)	4.8 (4.4–5.0)	0.08
Glucose (mg/dL)	47 (90–137)	47 (90–137)	41 (84.5–125.5)	0.3
NT-proBNP (pg/mL)	6198 (1518–7716)	3586 (1393.5–6322)	5863 (6519–12382)	0.007
hs-TnT (pg/mL)	40.9 (20.1–61)	30.0 (19.4–49.5)	35.9 (47.9–83.8)	0.0006

ATTR: transthyretin amyloid cardiomyopathy; eGFR: estimated glomerular filtration rate; hs-TnT: high-sensitivity cardiac troponin T; IVS: interventricular septum; LA: left atrium; LVEDD: left ventricular end-diastolic diameter; LVEF: left ventricular ejection fraction; LVESD: left ventricular end-systolic diameter; LVPW: left ventricular posterior wall; MDRD: modification of diet in renal disease; NT-proBNP: N-terminal pro b-type natriuretic peptide; RV: right ventricle; SGOT: serum glutamic oxaloacetic transaminase; SGPT: serum glutamate pyruvate transaminase.

**Table 3:** Short-term outcomes in the study population.

Outcomes	Total (n= 95)	Non-ATTR (n = 87)	ATTR (n = 8)
Deaths, n (%)	23 (24.2%)	17 (19.5%)	6 (75%)
Re-admissions, n (%)	28 (29.5%)	20 (23%)	8 (100%)

ATTR: transthyretin amyloid cardiomyopathy.

## Data Availability

Regarding the submission of raw data, in order to safeguard the protection of personal information, the data will be disclosed only in the event that reasonable requests are received. To request data from this study, the corresponding author should be contacted.
